# Molecular mechanisms of extracellular-ATP-mediated colorectal cancer progression: Implication of purinergic receptors-mediated nucleocytoplasmic shuttling of HuR

**DOI:** 10.1007/s11302-024-10021-2

**Published:** 2024-05-27

**Authors:** Abdel-Aziz S. Shatat, Elsayed M. Mahgoup, Mohammed H. Rashed, Ibrahim G. Saleh, El-Sayed Akool

**Affiliations:** 1 Department of Pharmacology and Toxicology, Faculty of Pharmacy, Al-Azhar University, Cairo, Egypt; 2 Department of Clinical Pharmacy, Faculty of Pharmacy, Al-Azhar University, Cairo, Egypt; 3 Department of Clinical Pharmacy and Pharmacy Practice, Faculty of Pharmacy, Sinai University, Kantra, Ismailia Egypt

**Keywords:** Colorectal cancer, P2R, ATP, HuR, Caco-2 cells

## Abstract

One of the leading causes of cancer-related deaths worldwide is colorectal cancer (CRC). Extracellular ATP (e-ATP) and purinergic receptors (P2R) play a central role in CRC proliferation and progression. Human antigen R (HuR) is becoming more and more understood to be essential for the expression of genes linked to cancer. The current study demonstrates that ATP can mediate CRC (Caco-2 cells) progression via induction of HuR nucleocytoplasmic shuttling and subsequent expression of cancer-related genes, a consequence mostly mediated via the P2R receptor. It was also noted that suppression of HuR activity by using dihydrotanshinone I (DHTS) prevents cancer-related gene expression and subsequent CRC (Caco-2 cells) progression induced by ATP. The expression of cyclin A2/cyclin-dependent kinase 2 (CDK2), Bcl-2, ProT-α, hypoxia‐inducible factor1-α (HIF1-α), vascular endothelial growth factor A (VEGF-A), transforming growth factor-β (TGF-β) and matrix metallopeptidase 9 (MMP-9) induced by ATP were highly reduced in the presence of either PPADS (non-selective P2R antagonist) or DHTS. In addition, e-ATP-induced Caco-2 cell proliferation as well as cell survival were highly reduced in the presence of either PPADS or DHTS or selective CDK-2 inhibitor (Roscovitine) or selective Bcl-2 inhibitor (ABT-263). Furthermore, it was found that MMP-9 is critical for Caco-2 cells migration induced by e-ATP as demonstrated by a clear reduction in cells migration in the presence of a selective MMP-9 inhibitor (Marimastat). Collectively, these data demonstrate that ATP through P2R activation can induce HuR nucleocytoplasmic shuttling that could be translated into an increase in cancer-related genes expression and subsequent, cell proliferation and progression.

## Introduction

Among the most prevalent tumors of the digestive system that has a serious impact on people’s physical and mental health is colorectal cancer (CRC) [1]. Although chemotherapy, radiation therapy, and surgery are the major forms of colorectal cancer (CRC) therapy that increases the overall survival rate of CRC patients, invasion and metastasis remain the leading causes of mortality for CRC patients [2]. For this reason, investigating the relevant molecular basis and pathogenic mechanisms influencing the pathogenesis and progression of CRC is crucial, to target the treatment of CRC, and to suppress the migration and metastasis of CRC.

Nucleotides and their plasma membrane receptors, purinergic receptors (P2R) have been shown to play a pivotal role in CRC proliferation and immune cell regulation [3–5]. Besides being a crucial component of energy information, adenosine 5′-triphosphate (ATP) also helps the body transmit signals, which are essential for regulating the life activities of all cells, including tumour cells. [6, 7]. Tumour cells have the ability to release significant amounts of ATP into the extracellular environment, and this molecule acts on other signalling molecules (such P2Y and P2X receptors) to regulate the growth of the tumour. [8]. Cancer cell growth, proliferation, apoptosis, metastasis, and invasion can all be controlled by activating P2R [9, 10]. Post-transcriptional mechanisms have been shown to have significant impacts on gene expression programs related to cancer. In several types of cancer, HuR, an RNA-binding protein, is highly prevalent. A large number of mRNAs regulated by HuR code for proteins linked to cancer development. Furthermore, HuR’s function in the expression of genes related to cancer is becoming increasingly evident. The function of HuR is based on its capacity to increase the synthesis of proteins that promote proliferation, suppress apoptosis, lower immune recognition, boost angiogenesis, and play a role in invasion and metastasis. Although HuR is mostly nuclear, its nuclear functions are still unknown. On the other hand, HuR’s translocation to the cytoplasm is closely related to its capacity to stabilize and/or regulate several target mRNAs translation [11]. It has been demonstrated that HuR binds to the 3′ UTR of cyclin A2 and stabilize the mRNA of cyclin A2 in CRC cells in a cell cycle-dependent manner, as cyclin A2, a cofactor of cyclin dependent kinase 2 (CDK-2) enhances tumor cells progression through the S phase [12]. In addition, increased cyclin A2 levels are linked to increased proliferation in tumors [13]. HuR has been shown to have the capacity to stabilize and regulate the translation of several mRNAs, which in turn inhibits the production of pro-apoptotic proteins and promotes the expression of anti-apoptotic proteins including B-cell lymphoma-2 (Bcl-2) [14] and Prothymosin α (ProTα) [15]. Tumour cells need to boost angiogenesis in order to expand [16]. Angiogenesis may be stimulated or inhibited by a variety of signals. It has been demonstrated that HuR has the ability to promote the expression of factors known to induce angiogenesis like Hypoxia-inducible factor-1α (HIF-1α) [17, 18] and vascular endothelial growth factor (VEGF) [19], and halting the production of anti-angiogenic factors.

It has been shown that TGF-β promotes tumour growth by allowing tumour cells to evade immune recognition [20]. HuR has been demonstrated to regulate TGF-β mRNA’s post-transcriptional expression in malignant brain tumors by binding its 3′UTR with great affinity [21]. Matrix metalloproteinase 9 (MMP-9) has been shown to cleave the majority of extracellular matrix (ECM) substrates in both healthy and pathological settings, including collagens, laminin, fibronectin, vitronectin, and proteoglycans [22]. High levels of invasion and/or metastasis of cervical, colorectal, gastric, pancreatic, breast, and oral cancers, as well as gliomas and skin tumors, are typically linked to MMP-9 expression, which is strongly expressed in a wide range of malignancies [23, 24]. HuR has been shown to bind with the 3′UTR of MMP-9 mRNA, stabilizing it and improving the production of the protein that is encoded [25].

Here, we hypothesize that large amounts of ATP can be released by tumor cells into the microenvironment. This ATP can regulate the growth of the tumour by acting on other signaling molecule (such as P2R) which in turn induces nucleocytoplasmic shuttling of HuR which increases the production of proteins linked to cancer, that promote angiogenesis, block apoptosis, boost proliferation, lower immune recognition, and facilitate invasion and metastasis. Therefore, the goal of this study was to elucidate the molecular mechanisms of ATP-mediated CRC progression to provide an entirely novel theoretical basis and data support for CRC therapy.

## Materials and methods

### Materials

ATP was obtained from Sigma-Aldrich (USA). A non-selective P2R antagonist, pyridoxalphosphate-6-azophenyl-2’,4’- disulfonic acid tetrasodium salt (PPADS) was obtained from Hello Bio Ltd. (UK). A selective HuR inhibitor, dihydrotanshinone I (DHTS), selective CDK-2 inhibitor, Roscovitine (Seliciclib), selective Bcl-2 inhibitor, ABT-263 (Navitoclax) and Selective MMP-9 inhibitor, Marimastat were obtained from Adooq Bioscience LLC (USA). Anti-HuR rabbit monoclonal antibody was from ABclonal (USA). Anti-Bcl-2 rabbit polyclonal antibody, anti-HIF1-α rabbit polyclonal antibody, anti-ProT-α rabbit polyclonal antibody and anti-VEGF-A rabbit polyclonal antibody were obtained from St John’s Laboratory Ltd (UK). β-actin monoclonal antibody was obtained from Cell Signaling Technology (USA). The goat anti-Rabbit IgG antibody was from Thermo-fisher scientific, Invitrogen (USA). DC protein assay (for protein quantification) was obtained from (Bio-Rad laboratories, USA). Human cyclin A2 ELISA Kit was from Cloud‐Clone Corp., Houston, TX, (USA). Human MMP-9 ELISA Kit was obtained from Bioassay Technology Laboratory Co., Ltd., (China). Human TGF-β_I_ and Human VEGF-A ELISA Kits were purchased from Elabscience Biotechnology Inc., (USA). Human Prothymosin- alpha (ProT-α; PTMA) ELISA Kit was obtained from Abbexa LLC, (USA). Human CDK-2 ELISA Kit was purchased from MyBioSource, San Diego, (USA). RIPA lysis buffer system was obtained from Santa Cruz Biotechnology (USA). Phosphate buffered saline (PBS) was obtained from Capricorn Scientific (Germany). Trypsin-Ethylenediaminetetraacetic acid (EDTA) was obtained from Capricorn Scientific (Germany).

### Cell culture

The adherent colorectal cancer cells (Caco-2 cells) were obtained from VACSERA, Egypt. From a 72-year-old White male patient with colorectal cancer, epithelial cells were isolated from colon tissue to create Caco-2 cells. Cells were cultured in RPMI 1640 (Roswell Park Memorial Institute) (Corning®, USA) supplemented with 100 U/ml of penicillin, 100 μg/ml of streptomycin (Pen-Strep) (Corning®, USA), and 10% foetal bovine serum (FBS) (Life Science Production, UK) and incubated in a humidified atmosphere with 5% CO2 at 37 °C.

### Indirect immunofluorescence microscopy

Nucleocytoplasmic shuttling of HuR mediated by ATP in CRC cells (Caco-2 cells) was analyzed by measuring the HuR levels in the cytoplasm by indirect immunofluorescence microscopy as previously described [26, 27]. Briefly, serum-free Caco-2 cells were incubated for 24 h at a confluence of around 70–80% before being stimulated with ATP or different inhibitors or ATP in combination with different inhibitors. After being rinsed with ice-cold phosphate-buffered saline (PBS), the cells were then incubated with methanol containing 0.02% (w/v) EDTA for 30 min at -20 °C. Following two PBS washes, the cells were blocked for one hour in PBS containing 3% (w/v) bovine serum albumin, and then they were incubated with anti-HuR rabbit monoclonal antibody (1:200) for two hours. After that, cells were repeatedly washed with PBS before being incubated for one hour with goat anti-rabbit IgG secondary antibody and then they washed with PBS again. Slides were mounted in Fluoromount G (Sigma Aldrich, USA) after staining the nuclei with DAPI (Sigma Aldrich, USA). A fluorescent microscope (Leica DM5500 B, Leica Microsystems, UK) was used to monitor the fluorescence of the cells. Using ImageJ/NIH software, each cultured cell’s fluorometric intensity was measured in at least 5–8 microscopic fields.

### Western blot analysis

Caco-2 cells were collected, rinsed with ice-cold PBS, and then lysed using RIPA lysis buffer. Following sonication and centrifugation, the supernatant was collected and divided into aliquots. The Bio-Rad DC Protein Assay was used to determine the total protein content in each sample [28]. Using 10% sodium dodecyl sulphate (SDS)-polyacrylamide gel electrophoresis, protein extracts were separated and then blotted polyvinylidene fluoride (PVDF) membranes (Millipore, USA) after denatured by Laemmli sample buffer. Then, membranes were blocked by incubating them for one hour at 4 °C in TBS buffer containing 0.1% Tween (TBST) and 5% BSA. After that, membranes were incubated overnight at 4 °C with the primary antibody. Following a TBST buffer wash, they were incubated with the secondary antibody for one hour at room temperature. After four TBST washes, Novex™ AP Chromogenic Substrate (BCIPTM NBT) was used to visualize the membrane-bound antibody-specific bands, and ImageJ/NIH software was used to quantify the band intensity.

### Measurement of cell proliferation

A colorimetric 50-bromo-2-deoxy-uridine (BrdU) labeling and detection kit III (Cell Biolabs, San Diego, USA) was used to measure cell proliferation in accordance with the manufacturer’s instructions [27]. In brief, 96-well plates were seeded with Caco-2 cells, and the cells were treated for 24 h with either vehicle, ATP, or ATP combined with various inhibitors. Afterwards, BrdU was added and the cells were labelled for 6 h at 37 °C. Immunological detection of BrdU was used to identify the incorporation of BrdU. A microplate reader was used to measure the extinction of the samples at 492 nm.

### Assessment of Cyclin A2, CDK-2, ProT-α, VEGF-A, MMP-9, and TGF-β expression using ELISA kits

Following the manufacturer’s instructions, Cyclin A2, CDK-2, ProT-α, VEGF-A, MMP-9, and TGF-β levels were determined using human ELISA Kits (Cloud‐Clone Corp., Houston, TX, USA), (MyBioSource, San Diego, USA), (Abbexa LLC, USA), (Elabscience Biotechnology Inc., USA), (Bioassay Technology Laboratory Co., Ltd., China) and (Elabscience Biotechnology Inc., USA), respectively.

### Cell viability assay

Cell viability was determined using the MTT assay. The Caco-2 cells (5000 cells/well) were transfected, and they were then cultured in 96-well plates and incubated at 37 °C. Following 24 and 48 h of incubation with ATP (in different concentrations), 100 µL of MTT (SERVA Electrophoresis GmbH, Germany) solution was added to wells (5mg/ml in PBS) and incubated at 37 °C for 4 h. The produced formazan crystals were then dissolved by adding 100µL of DMSO (Sigma, USA) to each well. The optical density was determined at 570 nm after the plates were gently shaken for ten minutes on a swing bed.

### Cell migration assay (Scratch Wound Healing Assay)

Using modified scratch wound healing assay, cell migration was assessed as previously described [29, 30]. Briefly, After 24 h of incubation, the old medium not changed. To inhibit cell proliferation, an optimized (nontoxic) dose of mitomycin C was added for confluent cells for a few hours and then mitomycin-C was removed by washing. A fresh 1 ml pipette tip was used to gently and slowly scratch the Caco-2 monolayer across the center of the well. Once the wells were scratched, the detached cells were removed from the wells by gently washing them twice with medium. The wells were replenished with fresh medium containing ATP alone or in combination with different inhibitors. Caco-2 cells were rinsed with 1 × PBS after 48 h. After that, 3.7% paraformaldehyde was used to fix the Caco-2 cells for 30 min. Following that, 1% crystal violet in 2% ethanol was used to stain the fixed Caco-2 cells for 30 min. A quantitative evaluation of the gap distance was performed using ImageJ/NIH software, and the rate of cell migration was calculated.

### Clonogenic Survival Assay (Colony formation assay)

Cell survival was evaluated as previously described [31, 32] using the colony formation assay. In brief, Caco-2 cells were seeded into 6-well plates and treated with either vehicle, ATP alone, or ATP combined with various inhibitors for 24 h. After 48 h of treatment, the wells were replenished with fresh medium. Then the colonies formed were rinsed with 10 ml PBS followed by fixation with acetic acid/methanol 1:7 (vol/vol) then staining with 1ml of 0.5% crystal violet solution at room temperature for 2 h. Then number of colonies were counted with a stereomicroscope. Then, efficiency and surviving fraction (SF) were calculated [33].

### Statistical analysis

The data are displayed as mean ± SD. One-way analysis of variance (ANOVA) was used for multiple comparisons, and Tukey–Kramer was used as a post-hoc test. *P* values less than 0.05 were the threshold for significance. The software GraphPad Prism version 5.01 was used to analyse the data.

## Results

### ATP enhanced Caco-2 cells viability in time- and dose-dependent manner

**Fig. 1:**
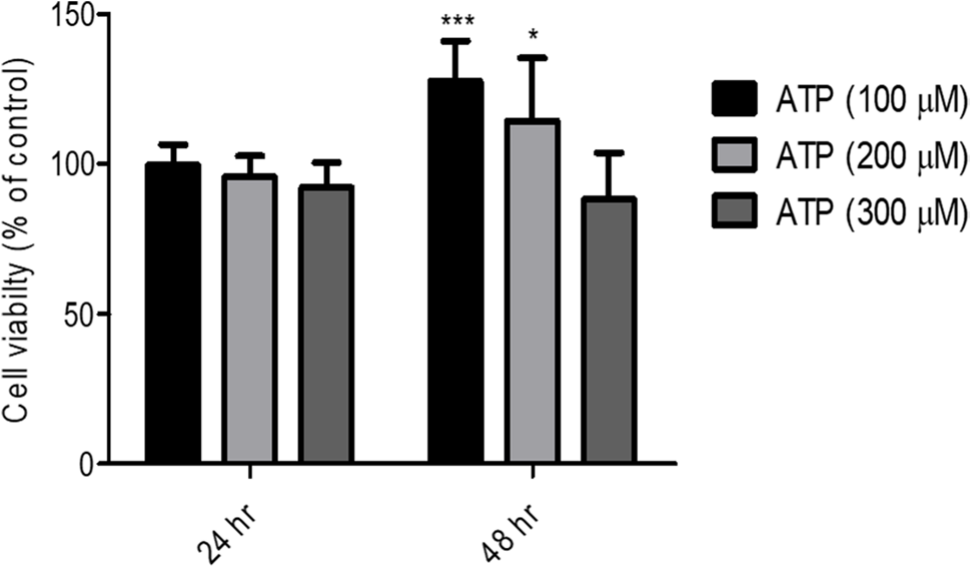
ATP enhanced cell viability in time- and dose-dependent manner. Caco-2 cells (CRC cells) were treated with either vehicle (Control) or ATP (100 μM, 200 μM and 300 μM) for 24 and 48 h. Viability of Caco-2 cells was determined using MTT assay. Data represent means ± S.D. (n = 10), ^***^Significantly different from control group at *p* < 0.001, ^*^Significantly different from control group at *p* < 0.05

First, the effect of ATP in different concentration (100 μM or 200 μM or 300 μM) on Caco-2 cells viability was examined. As demonstrated in Fig. 1, no significance difference in cells viability was observed after 24 h incubation with ATP. However, treatment of Caco-2 cells with ATP (100 μM or 200 μM) for 48 h significantly increased cells viability as compared to vehicle-treated cells (48 h).

### ATP-induced nucleocytoplasmic shuttling of HuR in Caco-2 cells in dose-dependent manner

**Fig. 2:**
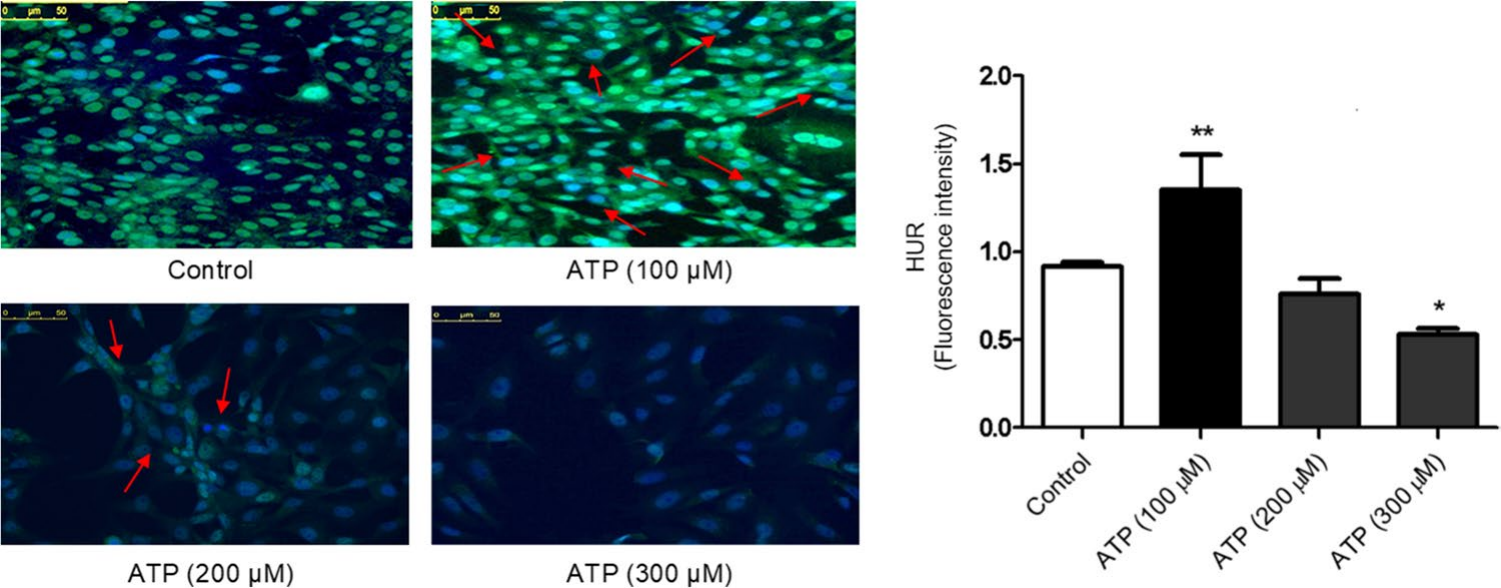
ATP-induced nucleocytoplasmic shuttling of HuR in dose-dependent manner. **A** CRC cells (Caco-2 cells) were treated with either vehicle (control) or ATP (100 μM, 200 μM and 300 μM) for 48 h and immunofluorescence analysis of HuR was performed (Scale bar: 0–50 µm). **B** The right panel shows fluorescence intensity of HuR. Data represent means ± SD. (n = 3), ^**^Significantly different from control at *p* < 0.01, ^*^Significantly different from control group at *p* < 0.05

To test the potential modulatory effect of ATP on nucleocytoplasmic shuttling of HuR, Caco-2 cells were treated with either vehicle or ATP (100, 200 or 300 μM) for 48 h. As demonstrated in Fig. 2, treatment of Caco-2 cells with ATP (100 μM) for 48 h significantly induced nucleocytoplasmic shuttling of HuR as indicated by an increase in cytoplasmic HuR fluorescence. However, no significant cytoplasmic HuR fluorescence was observed in Caco-2 cells stimulated with ATP (200 or 300 μM) for 48 h.

### Purinergic receptor is required for nucleocytoplasmic shuttling of HuR induced by ATP in Caco-2 cells

**Fig. 3:**
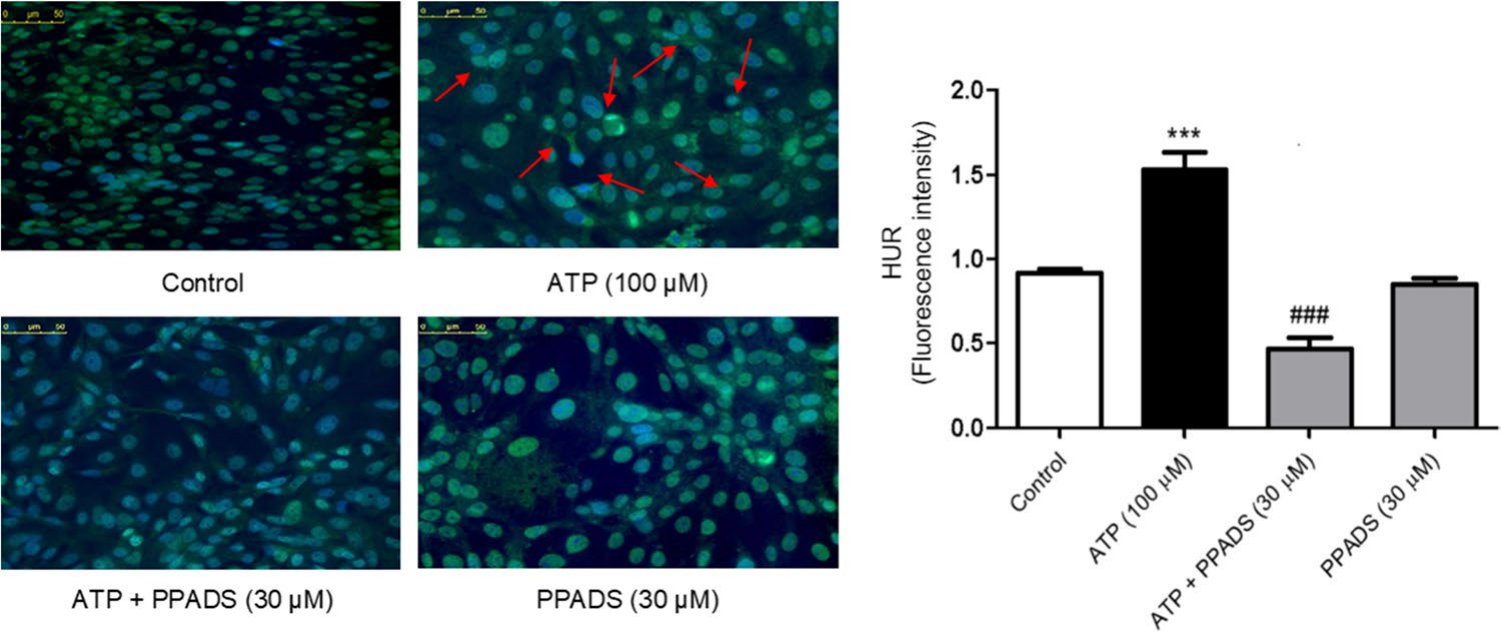
ATP-induced nucleocytoplasmic shuttling of HuR is abrogated in the presence of PPADS. Caco-2 cells were treated with either vehicle (control) or ATP or pretreated for 60 min with PPADS (non-selective P2R antagonist) before stimulation with ATP (100 μM) for 48 h and immunofluorescence analysis of HuR was performed (Scale bar: 0–50). The right panel shows fluorescence intensity of HuR. Data represent means ± SD. (n = 3), ^***^Significantly different from control at *p* < 0.001, and.^###^Significantly different from ATP-treated cells at *p* < 0.001

As demonstrated in Fig. 3, treatment of Caco-2 cells with ATP (100 μM) significantly induced nucleocytoplasmic shuttling of HuR. However, pre-incubation of Caco-2 cells with PPADS (non-selective P2R antagonist) for 60 min before treatment with either vehicle or ATP significantly inhibited the nucleocytoplasmic shuttling of HuR as indicated by nuclear trapping of HuR.

### Purinergic receptors activation and subsequent nucleocytoplasmic shuttling of HuR are required for ATP-induced cyclin A2/CDK-2 in Caco-2 cells

**Fig. 4:**
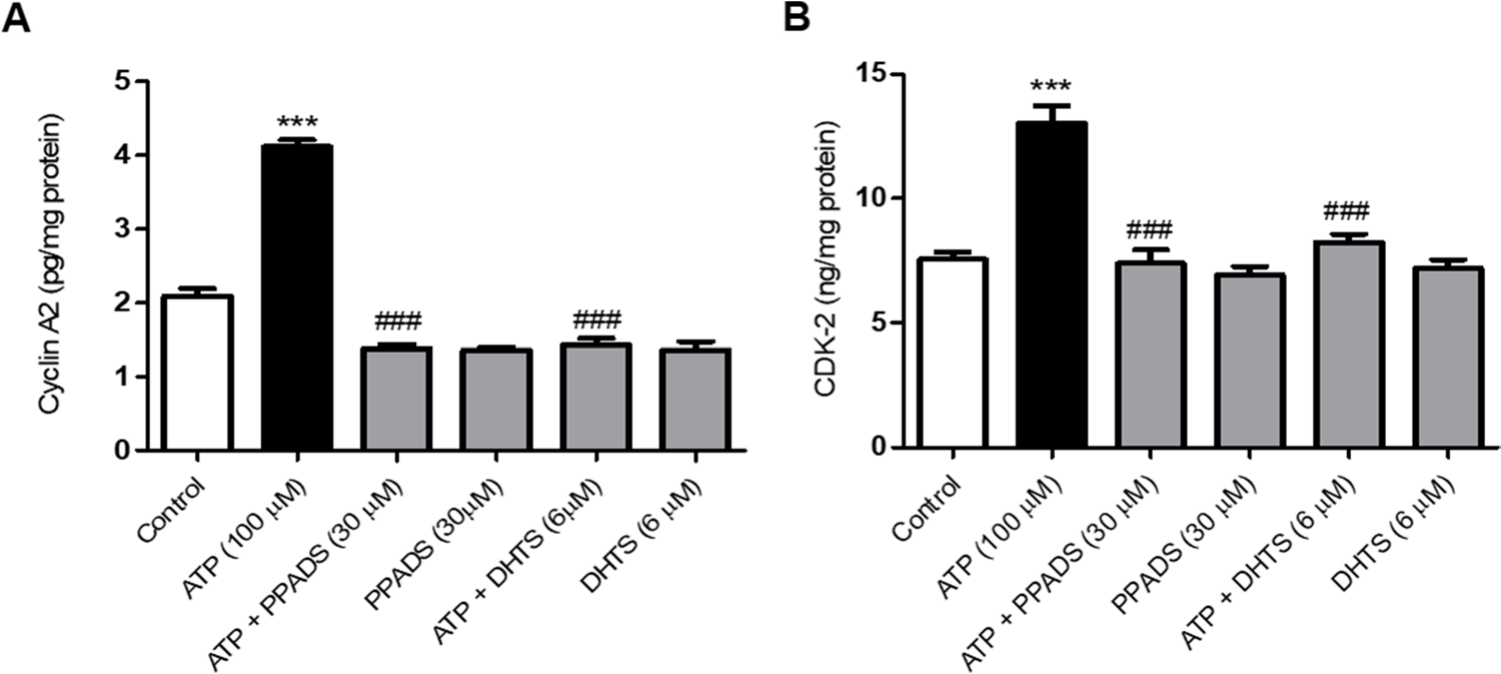
ATP-induced Cyclin A2 and CDK-2 expression in Caco-2 cells is abrogated in the presence of either PPADS or DHTS. Cyclin A2 (**A**) and CDK-2 (**B**) expression in Caco-2 cells treated with either vehicle (Control) or ATP (100 μM) or pretreated for 60 min with PPADS (non-selective P2R Antagonist) or DHTS (HuR inhibitor) before stimulation with ATP for 48 h were measured by ELISA. Data represent means ± S.D. (n = 3), ^***^Significantly different from control at *p* < 0.001, and.^###^Significantly different from ATP-treated cells at *p* < 0.001

As demonstrated in Fig. 4, treatment of Caco-2 cells with ATP significantly increased cyclin A2 and CDK-2 expression compared with control cells. In contrast, pre-incubation of Caco-2 cells with either PPADS or DHTS (HuR inhibitor) for 60 min before stimulation with ATP significantly decreased cyclin A2 as well as CDK-2 expression as compared to ATP alone-treated cells.

### Bcl-2 and ProT-α expression induced by ATP was attenuated in cells pre-incubated with either PPADS or DHTS

**Fig. 5:**
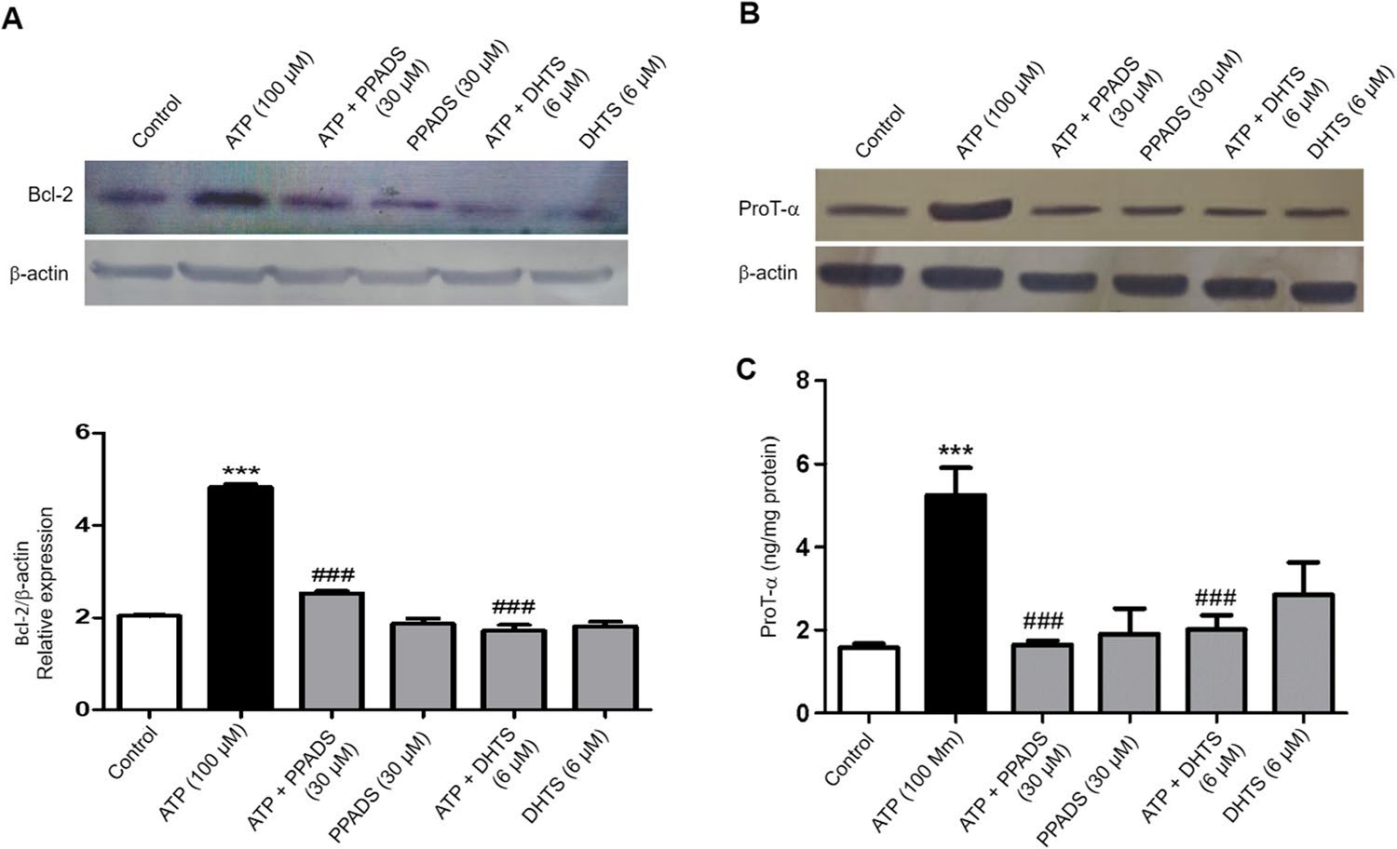
ATP-induced Bcl-2 and ProT-α expression, was attenuated in the presence of either PPADS or DHTS. Total cell extracts from Caco-2 cells treated with either vehicle (Control) or ATP (100 μM) or pretreated for 60 min with PPADS (non-selective P2R antagonist) or DHTS (HuR inhibitor) before stimulation with ATP for 48 h were subjected to western blot analysis and probed with either anti-Bcl-2 antibody (**A**) or anti-ProT-α antibody (**B**) or anti-β-actin antibody. The left lower panel shows a densitometric analysis of Bcl-2 in relation to β-actin level. **C** ProT-α expression in Caco-2 cells treated with either vehicle (Control) or ATP (100 μM) or pretreated for 60 min with PPADS or DHTS before stimulation with ATP for 48 h was measured using ELISA. Data represent means ± S.D. (n = 3), ^***^Significantly different from control at *p* < 0.001, and.^###^Significantly different from ATP-treated cells at *p* < 0.001

The expression of Bcl-2 and ProT-α was significantly induced in Caco-2 cells stimulated with ATP as compared to vehicle-treated cells (Fig. 5A, 5B). In contrast, pre-incubation of Caco-2 cells with either PPADS or DHTS for 60 min before stimulation with ATP significantly attenuated the expression of Bcl-2 and ProT-α as compared to ATP alone-treated cells (Fig. 5A, 5B).

### The expression of HIF1-α and VEGF-A in Caco-2 cells induced by ATP was abolished in cells pre-treated with either PPADS or DHTS

**Fig. 6:**
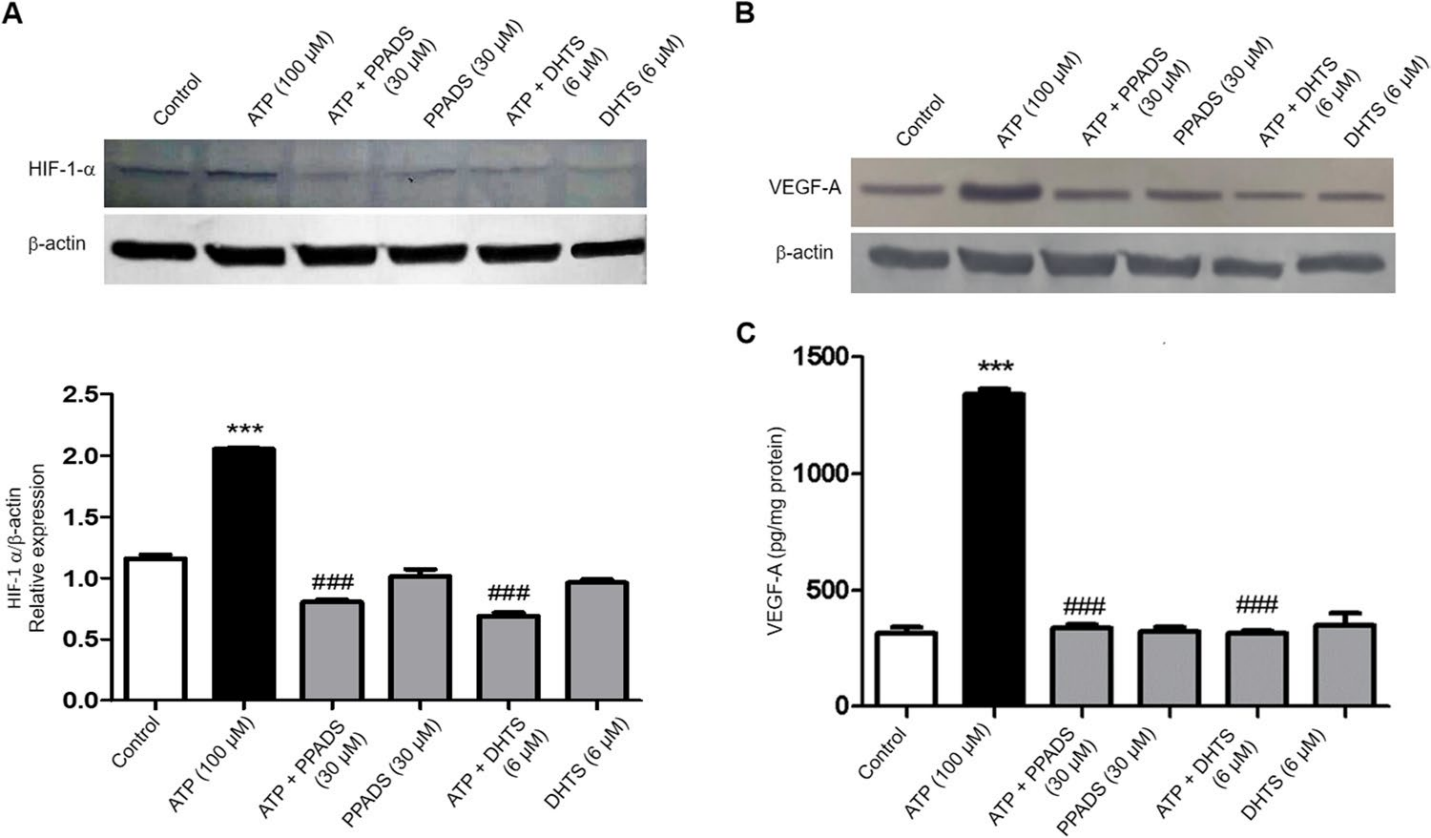
HIF1-α and VEGF-A expression induced by ATP was abrogated in the presence of either PPADS or DHTS. Total cell extracts from Caco-2 cells treated with either vehicle (Control) or ATP (100 μM) or pretreated for 60 min with PPADS (non-selective P2R antagonist) or DHTS (HuR inhibitor) before stimulation with ATP 100 for 48 h were subjected to western blot analysis and probed with either anti-HIF1-α antibody (**A**) or anti-VEGF-A antibody (**B**) or anti-β-actin antibody. The left lower panel shows a densitometric analysis of HIF1-α in relation to β-actin level. **C** The expression of VEGF-A in Caco-2 cells treated with either vehicle (Control) or ATP (100 μM) or pretreated for 60 min with PPADS or DHTS before stimulation with ATP for 48 h was measured using ELISA. Data represent means ± S.D. (n = 3), ^***^Significantly different from control at *p* < 0.001, and.^###^Significantly different from ATP-treated cells at *p* < 0.001

As shown in Fig. 6, stimulation of cells with ATP significantly induced the expression of HIF1-α and VEGF-A compared with vehicle-treated cells. However, pre-incubation of Caco-2 cells with either PPADS or DHTS for 60 min before stimulation with ATP significantly decreased HIF1-α and VEGF-A expression compared with ATP alone-treated cells.

### ATP-induced TGF-β and MMP-9 expression was attenuated in cells pre-incubated with either PPADS or DHTS

**Fig. 7:**
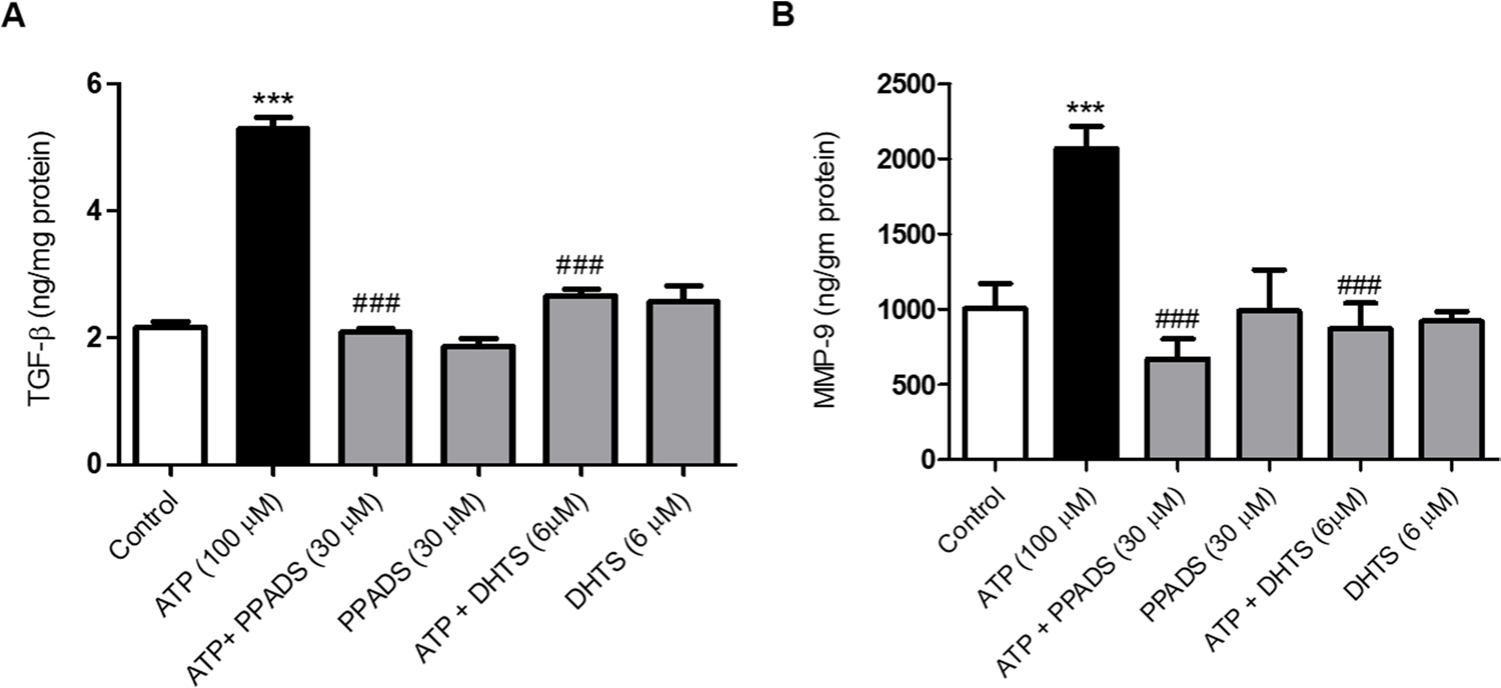
The expression of TGF-β and MMP-9 induced by ATP, was attenuated in the presence of either PPADS or DHTS. TGF-β (**A**) and MMP-9 (**B**) expression in Caco-2 cells treated with either vehicle (control) or ATP (100 μM) or pretreated for 60 min with PPADS (non-selective P2R antagonist) or DHTS (HuR inhibitor) before stimulation with ATP for 48 h was measured by ELISA. Data represent means ± S.D. (n = 3), ^***^Significantly different from control at *p* < 0.001, and.^###^Significantly different from ATP-treated cells at *p* < 0.001

Stimulation of Caco-2 cells with ATP significantly increased TGF-β (Fig. 7A) and MMP-9 (Fig. 7B) expression compared with control cells. However, pre-incubation of Caco-2 cells with either PPADS or DHTS for 60 min before stimulation with ATP, significantly decreased the expression of TGF-β (Fig. 7A) and MMP-9 (Fig. 7B) as compared to cells treated with ATP alone.

### ATP-induced Caco-2 cells migration was abrogated in the presence of either PPADS or DHTS or Marimastat

**Fig. 8:**
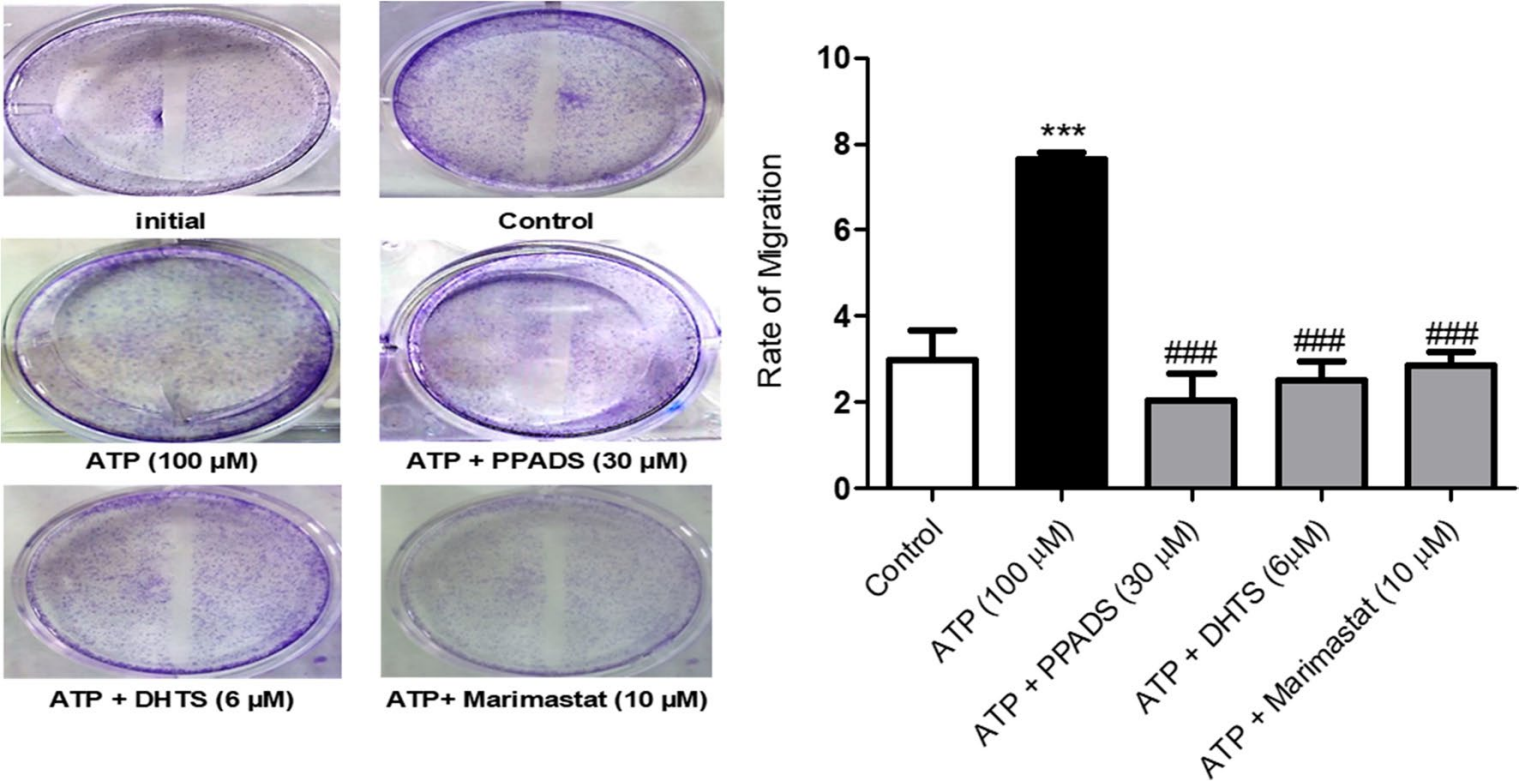
ATP-induced Caco-2 cells migration was abolished in cells pre-treated with either PPADS or DHTS or Marimastat. Caco-2 cells were stimulated with either vehicle (control) or ATP (100 μM) or pretreated for 60 min with Marimastat (Selective MMP-9 inhibitor) or PPADS (non-selective P2R antagonist) or DHTS (HuR inhibitor) before stimulation with ATP for 48 h, and the rate of migration was measured using scratch wound healing assay. Data represent means ± S.D. (n = 3), ^***^Significantly different from control at *p* < 0.001, and.^###^Significantly different from ATP-treated cells at *p* < 0.001

As demonstrated in Fig. 8, treatment of Caco-2 cells with ATP significantly increased the rate of migration compared with control cells. In contrast, pre-incubation of Caco-2 cells with either Marimastat (Selective MMP-9 inhibitor) or PPADS or DHTS for 60 min before stimulation with ATP significantly inhibited the migration of Caco-2 cells as compared to ATP alone-treated cells.

### PPADS, DHTS, and Roscovitine inhibited Caco-2 cells proliferation induced by ATP

**Fig. 9:**
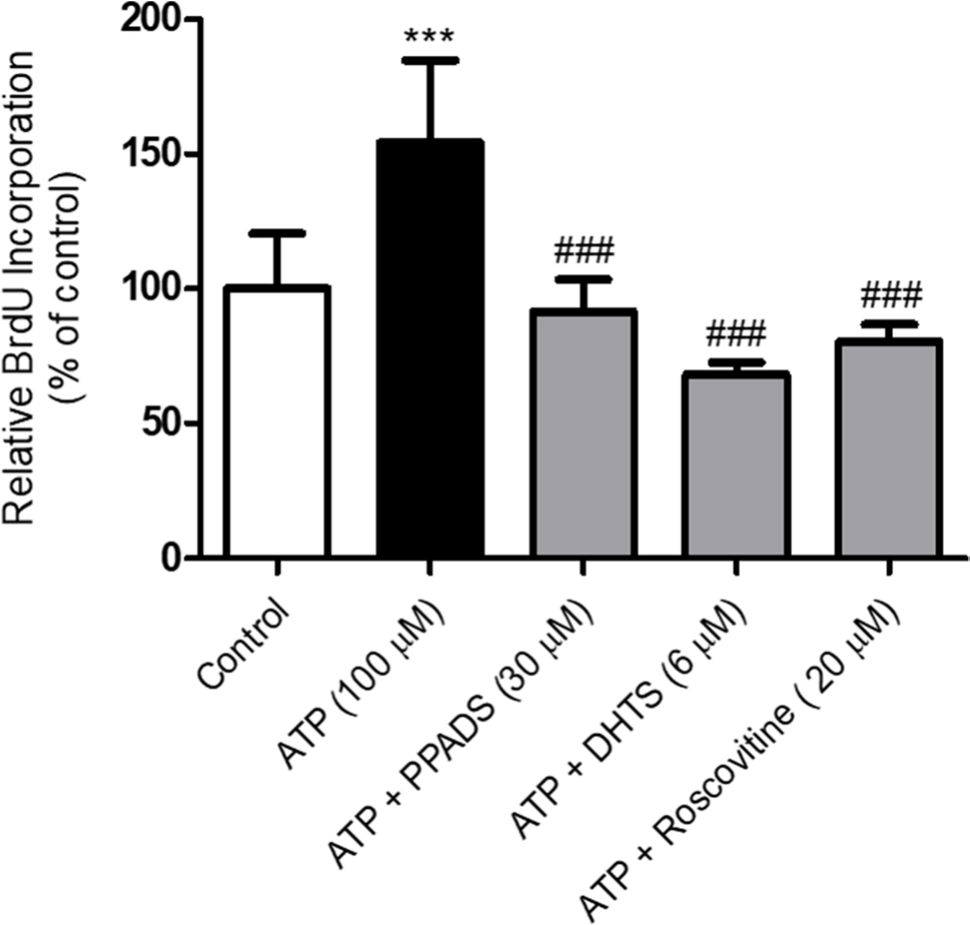
Caco-2 cells proliferation induced by ATP was abrogated in cells pre-treated with either PPADS or DHTS or Roscovitine. The proliferation of Caco-2 cells treated with either vehicle (control) or ATP (100 μM) or pretreated for 60 min with PPADS (non-selective P2R antagonist), DHTS (HuR inhibitor), Roscovitine (CDK2 inhibitor) before stimulation with ATP for 48 h was measured using BrdU labeling and detection kit. Data represent means ± S.D. (n = 3), ^***^Significantly different from control at *p* < 0.001, and.^###^Significantly different from ATP-treated cells at *p* < 0.001

To investigate whether the HuR nucleocytoplasmic shuttling induced by ATP would be functionally associated with an increase in Caco-2 cells proliferation, Caco-2 cells proliferation was measured using BrdU labeling and detection kit. As demonstrated in Fig. 9, treatment of Caco-2 cells with ATP significantly induced BrdU incorporation (cells proliferation) compared with control cells. However, pretreatment of Caco-2 cells with either PPADS or DHTS or Roscovitine (selective CDK-2 inhibitor) for 60 min before stimulation with ATP significantly decreased BrdU incorporation (cells proliferation).

### ABT-263, PPADS and DHTS inhibited Caco-2 cells survival induced by ATP

**Fig. 10:**
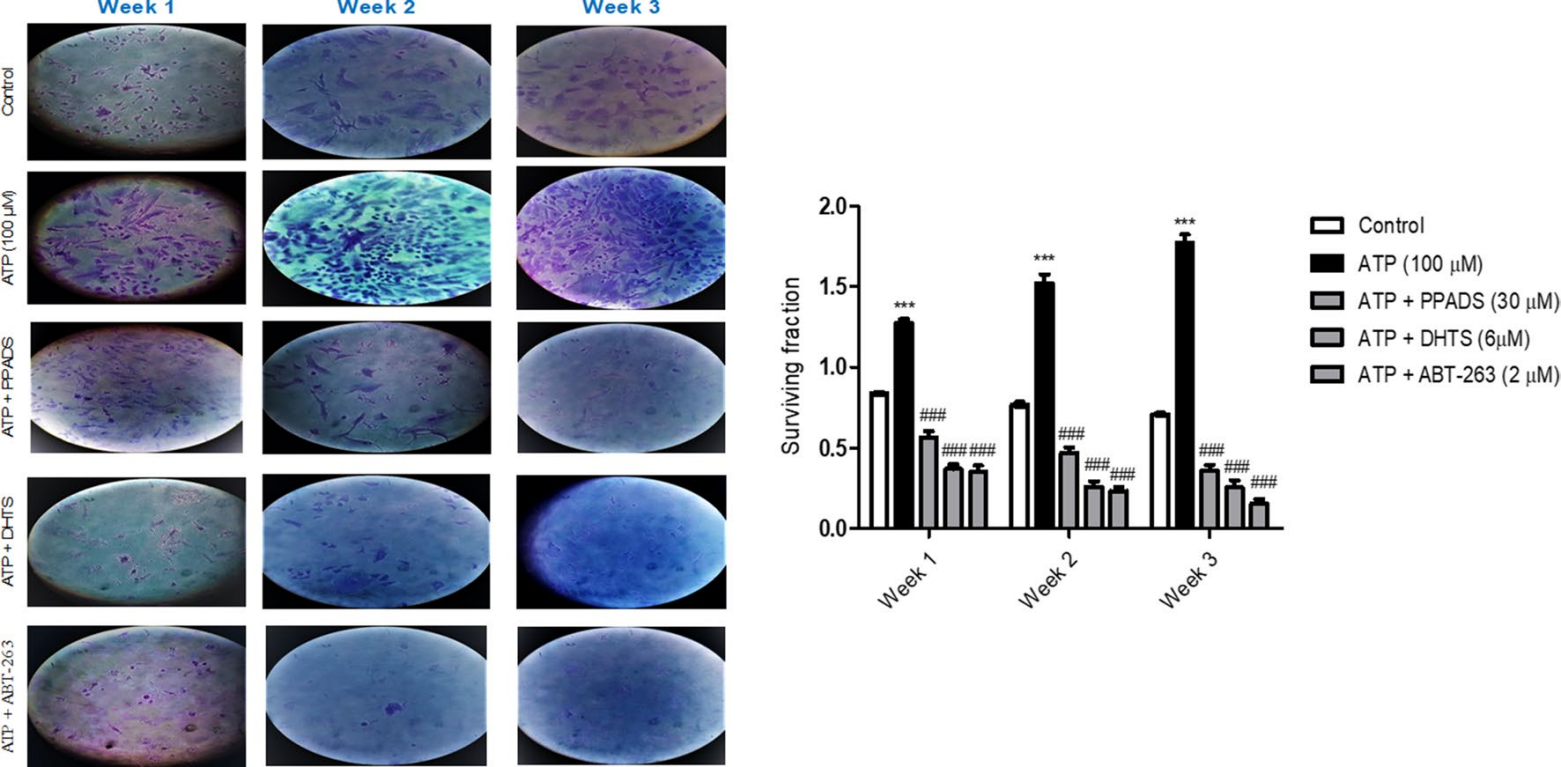
ATP-induced Caco-2 cells survival was attenuated in the presence of either ABT-263 or PPADS or DHTS. The surviving fraction of Caco-2 cells treated with either vehicle (control) or ATP (100 μM) or pretreated for 60 min with PPADS (non-selective P2R antagonist), DHTS (HuR inhibitor), or ABT-263 (Bcl-2 inhibitor) before stimulation with ATP for 48 h was measured using colony formation assay. Data represent means ± S.D. (n = 3), ^***^Significantly different from control at *p* < 0.001, and.^###^Significantly different from ATP-treated cells at *p* < 0.001

As demonstrated in Fig. 10, treatment of cells with ATP significantly increased the colony formation (Surviving fraction) as compared to control cells. However, pretreatment of Caco-2 cells with either PPADS or DHTS or ABT-263 (selective Bcl-2 inhibitor) for 60 min before stimulation with ATP significantly decreased the colony formation (Surviving fraction) as compared to ATP treated cells.

## Discussion 

Among the most prevalent gastrointestinal system tumors that has a serious impact on people’s physical and mental health is CRC. It is the third most frequent malignant tumor and the second most fatal cancer in the world [34]. Therefore, it was interesting to investigate the relevant molecular mechanisms involved in the pathogenesis and progression of CRC. It has been reported that nucleotides and their plasma membrane receptors, P2R are crucial for the development of CRC. Tumour cells have the capacity to release large amounts of ATP into the microenvironment, and this molecule acts on other signaling molecules (such as P2Y and P2X receptors) to regulate the growth of the tumour. [8]. Furthermore, ATP has been shown to be accumulated in tumor sites [35]. Previously, it has been demonstrated that post-transcriptional mechanisms regulate the expression of genes linked to cancer. It has been reported that the mRNA stabilizing factor, HUR is crucial for the post transcriptional regulation of the expression of cancer-related genes [36]. HuR performs this function by enhancing the mRNA stability, which then triggers the production of proteins that boost angiogenesis, suppress apoptosis, enhance proliferation, decrease immunological recognition, and enable invasion and metastasis [11]. HuR is primarily located in the nucleus. It shuttles between the cytoplasm and the nucleus, where the cytoplasm is widely believed to be the site of mRNA degradation [37]. This study presents the molecular mechanisms by which ATP-induced CRC progression. The current study demonstrates that ATP can induce nucleocytoplasmic shuttling of HuR in CRC cells (Caco-2 cells) through P2R receptors activation. These findings agree with earlier research demonstrated that ATP has the ability to induce nucleocytoplasmic shuttling of HuR in different cell types [25]. A functional role of P2R activation was demonstrated by a significant decline in HuR nucleocytoplasmic shuttling by using PPADS, a highly potent non-selective P2R antagonist. Cyclin A2/CDK-2, promotes cancer progression by driving the cell-cycle transition from S phase to G2 phase. It has been demonstrated that increased cell proliferation was linked to an increase in cyclin A2 levels in tumors [13]. In the current study, ATP increased cyclin A2/CDK-2 expression and subsequent cells proliferation. A functional involvement of P2R as well as HUR in cyclin A2/CDK-2 expression and subsequent cells proliferation was demonstrated by a significant decrease in cyclin A2/CDK-2 expression in the presence of either PPADS, or DHTS, a highly potent HuR inhibitor. These results are consistent with previous studies [12, 38, 39]. Furthermore, Caco-2 cells proliferation was significantly decreased in the presence of Roscovitine, selective CDK-2 inhibitor, indicating that CDK-2 is involved in CRC cells proliferation induced by ATP. The proto-oncogene Bcl-2 is a major anti-apoptotic protein that is expressed in a variety of malignancies and prevents the release of cytochrome c from the mitochondria [40]. In addition, ProTα prevents apoptosis by suppressing the formation of the apoptosome, a macromolecular complex that forms in cells undergoing apoptosis [41]. In accordance with those studies, Bcl-2 as well as ProTα were highly generated by ATP in Caco-2 cells. Most interestingly, Bcl-2 and ProTα expression as well as Caco-2 cells survival were markedly reduced in the presence of either PPADS or DHTS indicating that P2R/HUR are required for Bcl-2 and ProTα expression as well as Caco-2 cells survival induced by ATP. Furthermore, the functional involvement of Bcl-2 in cell survival and subsequent cells proliferation was highlighted by using ABT-263, a highly potent selective Bcl-2 inhibitor. These findings are consistent with a prior study demonstrating that ATP increases cell viability in lung cancer cells by regulating the cytosolic [Ca2 +] and Bcl-2/Bax ratios [42]. Many signals have the ability to either promote or prevent angiogenesis. It has been reported that HuR has the ability to promote the expression of factors known to induce angiogenesis like Hypoxia-inducible factor-1α (HIF-1α) [17, 18] and vascular endothelial growth factor (VEGF) [19]. Many studies have showed an elevation in HIF-1α in different kinds of tumors [43]. Also, it has been observed that HIF-1α levels are correlated with aggressive cancer characteristics, indicating that HIF-1α may be a target for cancer treatment [43]. Additionally, by promoting cell growth, angiogenesis, proliferation, and migration, VEGF enhances the development of cancer [44]. The current work demonstrates that ATP has the ability to increase HIF-1α and VEGF expression in Caco-2 cells. Importantly, this increase in HIF-1α and VEGF expression was abrogated in the presence of PPADS or DHTS indicating that P2R/HUR are critical for the expression of HIF-1α and VEGF. These findings are in agreement with previous findings [45, 46]. Previously, several research works have connected TGF-β’s carcinogenic potential to its ability to help cancer cells evade immune recognition [20]. Also, HuR has been demonstrated to control TGF-β mRNA’s post-transcriptional expression in malignant brain tumors by binding its 3′UTR with a high level of affinity [21]. This feature could be crucial to avoid immune recognition and enable the development of tumor. Tumour cells typically invade neighbouring tissues and spread to distant areas. It has been demonstrated that MMP-9 can cleave the majority of extracellular matrix (ECM) proteins in both physiological and pathological conditions, including collagen, laminin, fibronectin, vitronectin, and proteoglycans [22]. MMP-9 is highly induced in a wide range of malignancies and is often linked to increased invasiveness and/or metastasis in gliomas and skin tumors as well as colorectal, cervical, gastric, pancreatic, and breast cancers [23, 24]. Also, it has been shown that HuR interacts with MMP-9 mRNA’s 3′UTR to stabilize it and improve the production of the encoded protein [25]. In this study, TGF-β as well as MMP-9 were markedly increased in Caco-2 cells upon treatment with ATP. Interestingly, the expression of TGF-β and MMP-9 were highly reduced when the cells pre-treated with either PPADS or DHTS indicating that P2R/HUR are critical for TGF-β and MMP-9 expression induced by ATP in Caco-2 cells. Most interestingly, Caco-2 cell migration induced by ATP was highly decreased in cells pretreated with either PPADS or DHTS or Marimastat, selective MMP-9 inhibitor indicating that P2R/HUR/MMP-9 are required for Caco-2 cells migration induced by ATP. These findings are consistent with earlier studies [25, 47, 48].

## Conclusion

The current study shows for the first time that ATP through P2R activation can induce HuR nucleocytoplasmic shuttling that could be translated into an increase in cancer-related genes expression and subsequent, cell proliferation and progression. This study presents a mechanistic relevance of a signaling cascade involving ATP/P2R/HuR/cancer-associated protein expression and subsequent cancer progression. These data may provide a new theoretical basis and data support for CRC therapy. Targeting P2R/HUR could be a new approach for CRC treatment.

## Data Availability

All data and materials are available upon reasonable request.
